# Rocuronium Has a Suppressive Effect on Platelet Function via the P2Y12 Receptor Pathway In Vitro That Is Not Reversed by Sugammadex

**DOI:** 10.3390/ijms21176399

**Published:** 2020-09-03

**Authors:** Yutaka Murata, Shuji Kawamoto, Kazuhiko Fukuda

**Affiliations:** Department of Anesthesia, Kyoto University Hospital, 54 Shogoin Kawahara-cho, Sakyo-ku, Kyoto 606-8507, Japan; murata.yutaka.25a@st.kyoto-u.ac.jp (Y.M.); kfukuda@kuhp.kyoto-u.ac.jp (K.F.)

**Keywords:** rocuronium, sugammadex, morpholine, platelet, P2Y12 receptor, cyclodextrin

## Abstract

Rocuronium is an aminosteroid nondepolarizing neuromuscular blocker that is widely used for anesthesia and intensive care. In this study, we investigated the effect of rocuronium on human platelet functions in vitro. The effects of rocuronium on platelet aggregation, P-selectin expression, and cyclic adenosine monophosphate (cAMP) levels in platelets were measured using an aggregometer, an enzyme immunoassay, and flow cytometry, respectively. Rocuronium inhibited ADP-induced platelet aggregation, P-selectin expression and suppression of cAMP production. These effects were not antagonized by equimolar sugammadex, a synthetic γ-cyclodextrin derivative that antagonizes rocuronium-induced muscle relaxation by encapsulating the rocuronium molecule. Morpholine, which constitutes a part of the rocuronium molecule but is not encapsulated by sugammadex, inhibited ADP-induced platelet aggregation. Vecuronium, which has a molecular structure similar to that of rocuronium but does not possess a morpholine ring, had no significant effect on ADP-induced platelet aggregation. These results indicate that rocuronium has a suppressive effect on platelet functions in vitro that is not reversed by sugammadex and suggest that this effect is mediated by blockade of the P2Y12 receptor signaling pathway via the morpholine ring of rocuronium.

## 1. Introduction

Platelets are anucleate cells that play a central role in thrombus formation in blood. Therefore, information on the effects of perioperative drugs, including anesthetics, on platelet function is important for patient care. We have described the effects on platelets of various anesthetics used during the perioperative period [1,2,3,4,5,6,7], but the effects on human platelets of many drugs used perioperatively remain to be clarified. Rocuronium ((+)-(17β-acetoxy-3α-hydroxy-2β-morpholino-5α-androstan-16β-yl)-1-allyl-1-pyrrolidinium bromide), an aminosteroid nondepolarizing neuromuscular blocker, is widely used for anesthesia and intensive care [8], but its effect on human platelet functions has not been examined.

**Figure 1:**
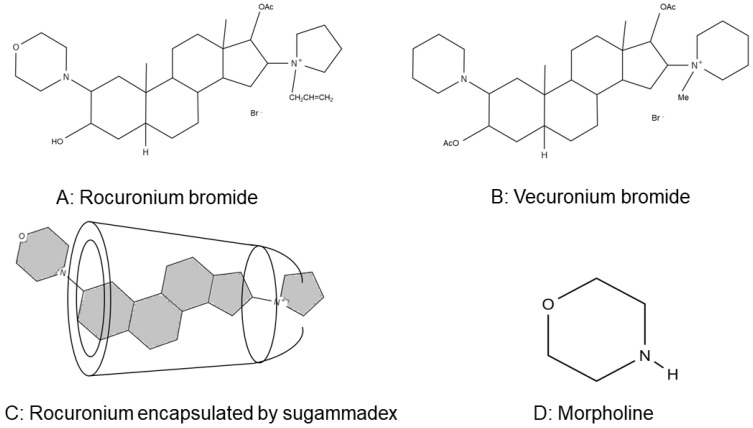
Structures of (**A**) rocuronium bromide; (**B**) vecuronium bromide; (**C**) rocuronium-sugammadex complex, based on a previous report [10]; (**D**) and morpholine.

Sugammadex, a cyclic oligosaccharide γ-cyclodextrin derivative, approved by the European Medicines Agency in 2008 and by the U.S. Food and Drug Administration in 2016 [9], encapsulates rocuronium to form a 1:1 complex in plasma and inhibits the binding of rocuronium with the nicotinic acetylcholine receptor expressed in the neuromuscular junction and reverses the neuromuscular blockade by rocuronium [10]. Sugammadex shows a similar effect against vecuronium, another widely used aminosteroid nondepolarizing neuromuscular blocker. The molecular structure of vecuronium resembles that of rocuronium but does not have a morpholine ring. The structures of rocuronium, vecuronium, the rocuronium/sugammadex complex, and morpholine are shown in Figure 1 [10].

Injury to blood vessels activates platelets locally via exposure of endothelial collagen. The activated platelets release dense granules that include adenosine diphosphate (ADP), which activates other platelets by autocrine or paracrine interactions. ADP binding to a platelet’s P2Y12 receptor inhibits adenylate cyclase and decreases the cyclic adenosine monophosphate (cAMP) level, which induces platelet activation by suppressing cAMP-dependent phosphorylation of the inositol triphosphate receptor and potentiating calcium release from the endoplasmic reticulum [11]. In this study, we investigated the effects of rocuronium on platelet functions activated by ADP and analyzed the mechanism of action.

## 2. Results

### 2.1. Platelet Aggregation

**Figure 2:**
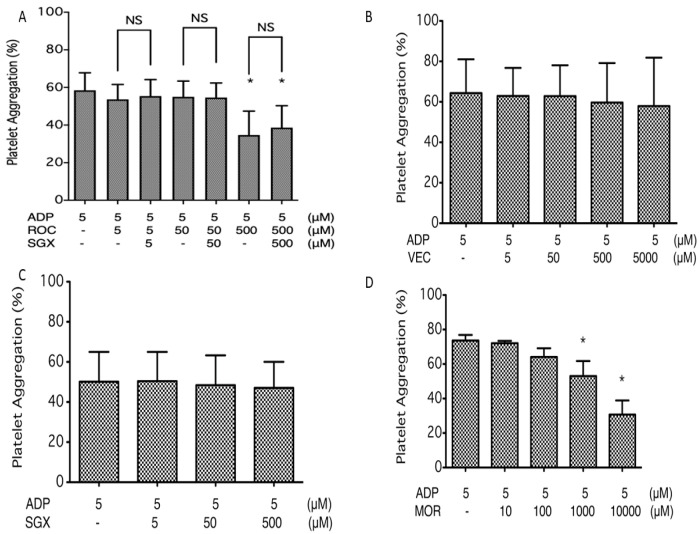
Effects of rocuronium, vecuronium, sugammadex, and morpholine on human platelet aggregation induced by 5 μM adenosine diphosphate (ADP). (**A**) Rocuronium (ROC) in the absence and presence of equimolar sugammadex (SGX) (5–500 μM). * *p* < 0.05 vs. ROC (−), SGX (−). (**B**) Vecuronium (VEC; 5–5000 μM). (**C**) Sugammadex (SGX; 5–500 μM). (**D**) Morpholine (MOR; 10 μM–10 mM). * *p* < 0.05 vs. MOR (−). Data are expressed as the mean ± SD. Rocuronium and morpholine suppressed ADP-induced platelet aggregation, whereas vecuronium and sugammadex did not show significant effects. The suppressive effect of rocuronium was not abolished by equimolar sugammadex.

Rocuronium at 500 μM suppressed platelet aggregation induced by 5 μM ADP (Figure 2A). In contrast, vecuronium (5–5000 μM) had no significant effect (Figure 2B). Sugammadex, which encapsulates rocuronium, did not affect ADP-induced platelet aggregation itself (Figure 2C) and did not significantly affect the suppressive effect of rocuronium (Figure 2A). The structural difference between rocuronium and vecuronium is the presence of a morpholine ring, which is not encapsulated by sugammadex (Figure 1C). Therefore, we next tested the effect of morpholine on platelet aggregation. Figure 2D shows that morpholine over 1 mM suppressed platelet aggregation induced by 5 μM ADP in a dose-dependent manner.

### 2.2. Flow Cytometry Analysis of P-Selectin Expression on ADP-Stimulated Platelets

**Figure 3:**
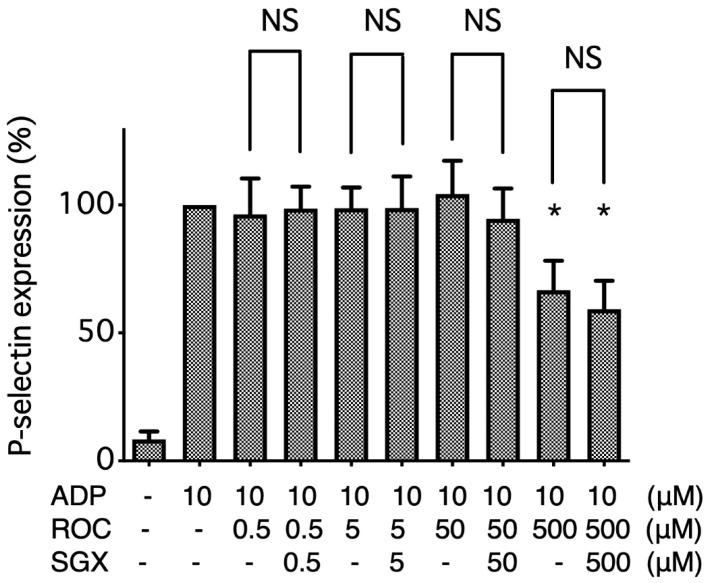
Effects of rocuronium (ROC; 0.5–500 μM) on surface P-selectin expression in platelets stimulated with 10 μM ADP in the presence and absence of equimolar sugammadex (SGX; 0.5–500 μM). Data are expressed as the mean ± SD of the ratio to the positive control (ROC (−), SGX (−), ADP (+)). * *p* < 0.05 vs. positive control. NS, not significant. Rocuronium suppressed P-selectin expression on the surface of ADP-stimulated platelets, and this was not significantly affected by equimolar sugammadex.

Rocuronium at 500 μM suppressed P-selectin expression on the surface of ADP-stimulated platelets. This effect was not abolished by equimolar sugammadex (Figure 3).

### 2.3. cAMP Formation

**Figure 4:**
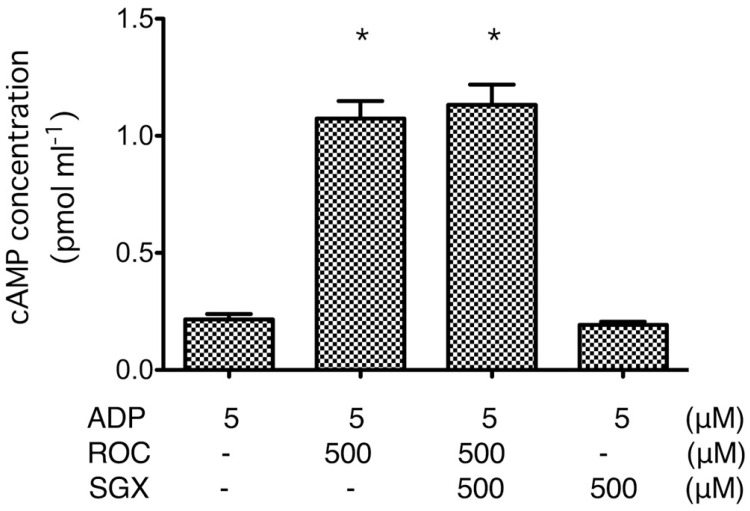
Effects of rocuronium (ROC; 500 μM) and sugammadex (SGX; 500 μM) on cyclic adenosine monophosphate (cAMP) formation in platelets stimulated with 5 μM ADP. Data are expressed as the mean ± SD. * *p* < 0.05 vs. ROC (−), SGX (−). Rocuronium reduced the suppression of cAMP production in ADP-stimulated platelets, which was not significantly affected by sugammadex. Sugammadex alone had no significant effect on cAMP formation.

Rocuronium at 500 μM increased cAMP production in ADP-stimulated platelets, and sugammadex had no influence on this effect. Sugammadex alone had no significant effect on cAMP formation (Figure 4).

## 3. Discussion

**Figure 5:**
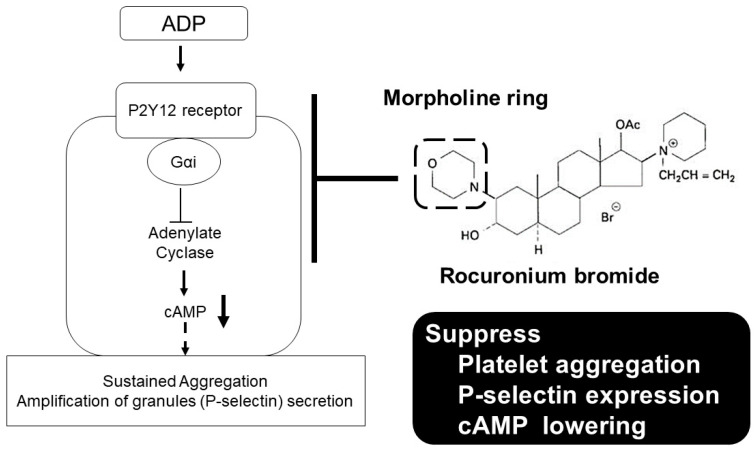
Proposed mechanism of the suppressive effect of rocuronium on platelet functions. Rocuronium suppresses platelet functions via its morpholine ring by blockade of the P2Y12 receptor signaling pathway. The morpholine ring in rocuronium is not encapsulated by sugammadex.

The main finding of this study was that rocuronium suppressed platelet functions, but the effect of rocuronium was not antagonized by sugammadex, an agent that reverses rocuronium-induced neuromuscular blockade. The suppressive action of rocuronium on platelets was suggested to be due to an increase in cAMP production caused by the inhibition of the P2Y12 receptor pathway by the morpholine ring in rocuronium, which was not encapsulated by sugammadex (Figure 5).

The P2Y12 receptor, a member of the P2Y family of purinergic G protein-coupled receptors, is mainly expressed on platelets [12]. When the P2Y12 receptor is activated by ADP, adenylate cyclase is inhibited by an inhibitory G protein and cAMP production from adenosine triphosphate decreases. The decline in cellular cAMP content reduces the phosphorylation level of the inositol trisphosphate receptor and causes platelet activation by calcium release from the endoplasmic reticulum [13], resulting in persistent platelet aggregation and secretion of granules, including P-selectin. In this study, rocuronium inhibited ADP-induced platelet aggregation and P-selectin expression and increased the platelet cAMP level in the presence of ADP, suggesting that rocuronium inhibits the P2Y12 receptor pathway and suppresses ADP-induced platelet activation. To further examine whether the inhibitory effect of rocuronium on platelet function is due to its antagonistic effect on the P2Y12 receptor, we should examine whether the effect of rocuronium is affected by the presence of P2Y12 receptor inhibitors, such as cangrelor or the active metabolite of prasugrel.

P-selectin (CD62P), a membrane protein present in the secretory granules (α granules) of platelets, is an adhesion molecule on the cell surface which is expressed in response to degranulation associated with cell activation, and is widely used as a marker for platelet activation and adhesive activity [14]. P-selectin stabilizes human platelet aggregates in the early phase in vitro and contributes to the enlargement and stabilization of arterial thrombi [15,16,17]. Our results suggest that a high concentration of rocuronium inhibits the adhesion of platelets, in which P-selectin participates, as well as platelet aggregation.

Morpholine is a heterocyclic amine with a structure in which the 1- and 4-carbon atoms in cyclohexane are replaced by nitrogen and oxygen, respectively (Figure 1D). Morpholine derivatives have diverse pharmacological actions, including antitumor, anti-inflammatory, and anti-parasitic effects [18,19,20,21,22,23]. Ahn et al. [24] identified six morpholine derivatives that suppressed platelet functions from 6211 substances using a light transmission aggregation test, and demonstrated that a morpholine derivative (6-[(4-dimethylamino-phenyl)-morpholin-4-yl-methyl]-benzo [1,3] dioxol-5-ol) acts directly and reversibly on a region of the P2Y12 receptor distinct from the binding site of a P2Y12 receptor antagonist, ticagrelor. However, it was not examined whether this morpholine derivative inhibited platelet functions through the interaction of its morpholine ring with the P2Y12 receptor. Our study shows that the inhibitory effects of rocuronium on ADP-induced platelet aggregation and P-selectin expression were not antagonized by equimolar sugammadex, which may suggest that rocuronium suppresses platelet functions via its morpholine ring, because this ring is not encapsulated by sugammadex [8,10]. This conclusion is consistent with the finding that ADP-induced platelet aggregation was not inhibited by vecuronium, which does not have a morpholine ring. Furthermore, the suppressive effect of morpholine itself on ADP-induced platelet aggregation also supports this conclusion. It might be possible that other morpholine derivatives, such as levofloxacin, linezolid, and gefitinib, suppress platelet functions via their morpholine ring [18].

The influence of aminosteroid neuromuscular blockers, such as rocuronium and vecuronium on intraoperative hemorrhages, has not been reported. The blood rocuronium level rose to 50 μM immediately after injection of the clinical dosage for tracheal intubation (0.9 mg/kg), and the blood level was maintained above 5 μM for about 30 min [25]. The blood rocuronium level is significantly higher during surgery in patients with hepatic and renal dysfunctions compared to patients with normal hepatic and renal functions [26], because rocuronium is mainly excreted into bile in the liver, and partially excreted in urine [27,28]. In elderly patients, the blood rocuronium level might be higher than in young patients when the same dose of rocuronium is administered because elimination of rocuronium slows with age [29]. Thus, the blood rocuronium level in some clinical situations may be higher than the typical level in healthy people. However, it is unlikely that rocuronium concentrations would reach 500 μM in standard clinical use. In clinical practice, rocuronium concentrations above 500 μM are only likely to be reached in the case of an error in administration. The most likely situation would be when adult doses are given to pediatric patients. In these situations, rocuronium may suppress platelet functions and induce perioperative bleeding.

Cyclodextrins are cyclic oligosaccharides assembled from D-(+) glucose subunits connected by α-(1,4) glycosidic bonds to form torus-like macro rings. Cyclodextrin derivatives are used in food, pharmaceuticals, drug delivery, and the chemical industry, as well as in agriculture and environmental engineering [9,30], mainly as inert excipients to improve the stability and solubility of molecules. Some cyclodextrin derivatives, such as sugammadex, can inhibit the effect of an encapsulated drug, and drugs encapsulated by cyclodextrin derivatives generally show no pharmacological activity. Although it has not been examined whether rocuronium encapsulated by sugammadex retains its neuromuscular blocking effect [10,31], to the best of our knowledge, this study is the first to show that a drug encapsulated by a cyclodextrin derivative can retain a pharmacological effect.

There are several limitations of this study. First, although the results suggest that rocuronium has an inhibitory effect on the P2Y12 receptor signal transduction pathway, it remains to be clarified which molecule in the signal transduction pathway is directly affected by rocuronium. Because rocuronium has a steroid skeleton, it may pass through the cell membrane and directly activate adenylate cyclase. Moreover, in addition to the P2Y12 receptor, the P2Y1 receptor on platelets can be activated by ADP, which then activates phospholipase C via Gq, causing an increase in intracellular calcium ions, platelet shape-change, and transient platelet aggregation [11]. We have focused on the P2Y12 receptor pathway as the point of action of rocuronium, but we cannot exclude the possibility that rocuronium can also affect the P2Y1 pathway. We measured cAMP as a component of the P2Y12 pathway, but the cAMP level is also regulated by mechanisms other than the P2Y12 receptor pathway, including the α_2_-adrenoceptor pathway, and their influences cannot be ruled out [32]. The effects of rocuronium on platelets when they are stimulated by activators other than ADP also need to be studied. Furthermore, we should evaluate the other parameters (e.g., mobilization of intracellular calcium, phosphorylation of vasodilator-stimulated phosphoproteins, and activity of specific kinases) in future studies. Second, because platelet-rich plasma (PRP), not whole blood, was used in the aggregation tests and P-selectin expression measurements, the influence of any interaction between the platelets and the other blood cells on the effect of rocuronium could not be evaluated. As a next step, we need to confirm the inhibitory effect of rocuronium on platelet function using in vivo models. Third, we should have investigated whether a substance with the structure of rocuronium without the morpholine ring has an antiplatelet effect. However, because such a substance is not currently available on the market, and because it would be time-consuming to synthesize one by ourselves, we alternatively used vecuronium, which has a similar structure to rocuronium but without the morpholine ring. Within these limitations, this study shows that rocuronium has a suppressive effect on platelet functions in vitro, and that this effect is mediated by the blockade of the P2Y12 receptor signaling pathway via the morpholine ring of rocuronium.

## 4. Materials and Methods

### 4.1. Platelet Preparation

This study was approved by the ethics committee of Kyoto University Hospital (R0978; 21 March 2017). Prior written informed consent was obtained from the subjects. Venous blood was obtained by venipuncture of the forearm veins of 15 healthy volunteers who had not taken any medication for at least two weeks before the blood sampling. The blood was mixed with a 10% volume of 3.8% trisodium citrate. The number of platelets was counted using an automated hematology analyzer (KX-21N Sysmex America, Mundelein, IL, USA). The blood sample was centrifuged at 160× *g* for 10 min at room temperature and the supernatant was collected as platelet-rich plasma (PRP). The remaining lower portion of the first centrifuged blood sample was further centrifuged at 1600× *g* for 30 min at room temperature, and the clear supernatant was used as platelet-poor plasma (PPP). The platelet count was adjusted to 3 × 10^5^/μL by dilution with PPP (adjusted PRP). Washed platelets for the cAMP assay were prepared from PRP. A 10% volume of 100 mM ethylenediaminetetraacetic acid (EDTA) was added to adjusted PRP. After 15 min centrifugation at 900× *g*, the platelet pellet was gently suspended in a wash buffer (pH 7.39) containing 2 mM NaH_2_PO_4_, 8 mM Na_2_HPO_4_, 10 mM EDTA, 5 mM KCl, and 135 mM NaCl at 4 °C. The platelet pellet was centrifuged again at 900× *g* for 15 min at 4 °C and finally suspended as washed platelets in an assay buffer (pH 7.96) containing 10 mM *N*-2-hydroxyethylpiperazine-*N*′-2′-ethanesulfonic acid (HEPES), 0.5 mM Na_2_HPO_4_, 6 mM glucose, 5 mM KCl, and 145 mM NaCl. The platelet count in the final suspension was adjusted to 10^6^/μL with the same buffer.

### 4.2. Chemicals and Drugs

Rocuronium bromide, vecuronium bromide, and morpholine were purchased from Nacalai Tesque (Kyoto, Japan). Sugammadex sodium was purchased from MSD (Tokyo, Japan). ADP was purchased from Sigma-Aldrich (St. Louis, MO, USA). Peridinin–chlorophyll–protein (PerCP)-labeled anti-CD61 antibody, phycoerythrin (PE)-labeled anti-CD62P (P-selectin) antibody, and PE-labeled IgG as controls were obtained from Becton Dickinson (San Diego, CA, USA). All other chemicals were of analytical grade. We confirmed that all of the buffers or solvents used for dilution in our experiments had no effects on the results.

### 4.3. Measurement of ADP-Induced Platelet Aggregation

Aggregation induced by ADP was measured with an aggregometer (MCM Hema Tracer 212; MC Medical, Tokyo, Japan). Adjusted PRP (3 × 10^5^/μL, 200 μL) was pipetted into a cylindrical cuvette containing rocuronium, vecuronium, or morpholine in the presence or absence of sugammadex and incubated at 37 °C for 3 min. Then, the sample was stirred at 37 °C with a magnetic bar at 1000 rpm. A 5 μL volume of 200 μM ADP (final concentration: 5 μM) was added to the cylindrical cuvette, and ADP-induced aggregation was measured for 7 min as a change in light transmission from that of PPP, which was taken to be 100%.

### 4.4. Flow Cytometry Analysis of P-Selectin Expression on ADP-Stimulated Platelets

Flow cytometry was performed as we have described previously [6]. Adjusted PRP was diluted 10-fold with phosphate-buffered saline (PBS) (pH 7.42) containing 139 mM NaCl, 8.1 mM NaHPO_4_, 1.5 mM KH_2_PO_4_, and 2.7 mM KCl. For ADP-stimulated platelet analysis, an aliquot of adjusted PRP was incubated with rocuronium in the presence or absence of sugammadex at room temperature for 3 min. ADP (final concentration: 10 μM) was then added and incubated for 30 min. The samples were fixed with ice-cold 1% formaldehyde for 60 min on ice and washed twice with ice-cold PBS by centrifugation at 900× *g* for 15 min at 4 °C. The pellet was suspended in 100 μL PBS at 4 °C. Then, 20 μL of the platelet suspension was coincubated with PerCP-labeled anti-CD61 antibody and PE-labeled anti-CD62P (P-selectin) antibody in a final volume of 100 μL adjusted with PBS for 60 min at room temperature in the dark. PE-labeled IgG was used to estimate nonspecific binding. The reaction was stopped by adding ice-cold PBS. Samples were analyzed using a fluorescence-activated cell sorting (FACS) Calibur instrument (Becton Dickinson, San Jose, CA, USA). For each sample, data from 10,000 platelets were collected. Platelets were identified by forward and side scatter intensity and by CD61 expression. P-selectin levels on activated platelet surface membranes were recorded as the mean fluorescent intensity (MFI) of PE.

### 4.5. cAMP Assay

Washed platelets were used in the cAMP assays to exclude possible effects of other blood cells on the results. A platelet suspension (100 μL) was incubated at 37 °C for 3 min in a cylindrical cuvette in the presence or absence of 500 μM rocuronium with or without 500 μM sugammadex, and then stirred with a magnetic bar at 1000 rpm at 37 °C for 7 min with stimulation by 5 μM ADP. The reaction was terminated by adding a 10% volume of 0.1 M ice-cold HCl. The cAMP measurements were performed using commercial enzyme immunoassay kits (Cyclic AMP EIA Kit No. 581,001 Cayman Chemical, Ann Arbor, MI, USA), according to the manufacturer’s protocol.

### 4.6. Statistical Analysis

All data are expressed as the mean ± standard deviation (SD) of three to six separate experiments. Group variances were tested by a Brown-Forsythe test, and were statistically equal. All data were compared by one-way ANOVA, followed by a Dunnett test. All analyses were performed using Prism 5.0 software (GraphPad Inc., La Jolla, CA, USA) with *p* < 0.05 considered significant.

## 5. Conclusions

This study shows that rocuronium has a suppressive effect on platelet functions in vitro. This effect is mediated by the blockade of the P2Y12 receptor signaling pathway via the morpholine ring of rocuronium.

## Abbreviations

ADP	Adenosine diphosphate
cAMP	Cyclic adenosine monophosphate
EDTA	Ethylenediaminetetraacetic acid
FACS	Fluorescence-activated cell sorting
HEPES	*N*-2-hydroxyethylpiperazine-*N*′-2′-ethanesulfonic acid
MFI	Mean fluorescent intensity
MOR	Morpholine
PBS	Phosphate-buffered saline
PE	Phycoerythrin
PerCP	Peridinin Chlorophyll Protein
PPP	Platelet-poor plasma
PRP	Platelet-rich plasma
ROC	Rocuronium
SGX	Sugammadex
VEC	Vecuronium

## References

[1] Hirakata H., Hatano Y., Ushikubi F., Mori K., Narumiya S., Nakamura K. (1995). The Effect of Inhaled Anesthetics on the Platelet Aggregation and the Ligand-Binding Affinity of the Platelet Thromboxane A2 Receptor. Anesth. Analg..

[2] Hirakata H., Ushikubi F., Toda H., Nakamura K., Sai S., Urabe N., Hatano Y., Narumiya S., Mori K. (1996). Sevoflurane inhibits human platelet aggregation and thromboxane A 2 formation, possibly by suppression of cyclooxygenase activity. Anesthesiology.

[3] Hirakata H., Nakamura K., Sai S., Okuda H., Hatano Y., Urabe N., Mori K. (1997). Platelet aggregation is impaired during anaesthesia with sevoflurane but not with isoflurane. Can. J. Anaesth..

[4] Hirakata H., Nakamura K., Yokubol B., Toda H., Hatano Y., Urabe N., Mori K. (1999). Propofol Has Both Enhancing and Suppressing Eflects on Human Platelet Aggregation. Anesthesiology.

[5] Nakagawa T., Hirakata H., Sato M., Nakamura K., Hatano Y., Nakamura T., Fukuda K. (2002). Ketamine suppresses platelet aggregation possibly by suppressed inositol triphosphate formation and subsequent suppression of cytosolic calcium increase. Anesthesiology.

[6] Kawamoto S., Hirakata H., Sugita N., Fukuda K. (2015). Bidirectional effects of dexmedetomidine on human platelet functions in vitro. Eur. J. Pharmacol..

[7] Kawamoto S., Fukuda K. (2018). Dexmedetomidine increases human platelet-derived microparticles via the α2-adrenoceptor. J. Jpn. Soc. Intensive Care Med..

[8] Hunter J.M. (1996). Rocuronium: The newest aminosteroid neuromuscular blocking drug. Br. J. Anaesth..

[9] Braga S.S. (2019). Cyclodextrins: Emerging Medicines of the New Millennium. Biomolecules.

[10] Welliver M., McDonough J., Kalynych N., Redfern R. (2008). Discovery, development, and clinical application of sugammadex sodium, a selective relaxant binding agent. Drug Des. Dev. Ther..

[11] Kahner B.N., Shankar H., Murugappan S., Prasad G.L., Kunapuli S.P. (2006). Nucleotide receptor signaling in platelets. J. Thromb. Haemost..

[12] Gachet C. (2008). P2 receptors, platelet function and pharmacological implications. Thromb. Haemost..

[13] Keularts I.M.L.W., van Gorp R.M.A., Feijge M.A.H., Vuist W.M.J., Heemskerk J.W.M. (2000). α 2A-Adrenergic Receptor Stimulation Potentiates Calcium Release in Platelets by Modulating cAMP Levels. J. Biol. Chem..

[14] Pabinger I., Thaler J., Ay C. (2013). Biomarkers for prediction of venous thromboembolism in cancer. Blood.

[15] Merten M., Thiagarajan P. (2000). P-Selectin Expression on Platelets Determines Size and Stability of Platelet Aggregates. Circulation.

[16] Christersson C., Johnell M., Siegbahn A. (2008). Tissue factor and IL8 production by P-selectin-dependent platelet-monocyte aggregates in whole blood involves phosphorylation of Lyn and is inhibited by IL10. J. Thromb. Haemost..

[17] Yokoyama S., Ikeda H., Haramaki N., Yasukawa H., Murohara T., Imaizumi T. (2005). Platelet P-selectin plays an important role in arterial thrombogenesis by forming large stable platelet-leukocyte aggregates. J. Am. Coll. Cardiol..

[18] Naim M.J., Alam O., Alam M.J., Alam P., Shrivastava N. (2015). A review on pharmacological profile of Morpholine derivatives. Int. J. Pharmacol. Pharm. Sci..

[19] Wang X.M., Xin M.H., Xu J., Kang B.R., Li Y., Lu S.M., Zhang S.Q. (2015). Synthesis and antitumor activities evaluation of m-(4-morpholinoquinazolin-2-yl)benzamides in vitro and in vivo. Eur. J. Med. Chem..

[20] Senwar K.R., Sharma P., Reddy T.S., Jeengar M.K., Nayak V.L., Naidu V.G.M., Kamal A., Shankaraiah N. (2015). Spirooxindole-derived morpholine-fused-1,2,3-triazoles: Design, synthesis, cytotoxicity and apoptosis inducing studies. Eur. J. Med. Chem..

[21] Smelcerovic A., Rangelov M., Smelcerovic Z., Veljkovic A., Cherneva E., Yancheva D., Nikolic G.M., Petronijevic Z., Kocic G. (2013). Two 6-(propan-2-yl)-4-methyl-morpholine-2,5-diones as new non-purine xanthine oxidase inhibitors and anti-inflammatory agents. Food Chem. Toxicol..

[22] Khanum S.A., Begum B.A., Girish V., Khanum N.F. (2010). Synthesis and evaluation of benzophenone-n-ethyl morpholine ethers as anti-inflammatory agents. Int. J. Biomed. Sci..

[23] Kuettel S., Zambon A., Kaiser M., Brun R., Scapozza L., Perozzo R. (2007). Synthesis and evaluation of antiparasitic activities of new 4-[5-(4-phenoxyphenyl)-2H-pyrazol-3-yl]morpholine derivatives. J. Med. Chem..

[24] Ahn Y.H., Lee J.Y., Park H.D., Kim T.H., Park M.C., Choi G., Kim S. (2016). Identification of a new morpholine scaffold as a P2Y12 receptor antagonist. Molecules.

[25] Suzuki T., Saeki S., Takeda J., Ozaki M., Iwao Y. (2006). Neuromuscular blocking effects, pharmacokinetics and safety of Org 9426 (rocuronium bromide) in Japanese patients. Masui.

[26] Bevan D.R. (1994). Rocuronium bromide and organ function. Eur. J. Anaesthesiol. Suppl..

[27] Magorian T., Wood P., Caldwell J., Fisher D., Segredo V., Szenohradszky J., Sharma M., Gruenke L., Miller R. (1995). The Pharmacokinetics and Neuromuscular Effects of Rocuronium Bromide in Patients with Liver Disease. Anesth. Analg..

[28] Szenohradszky J., Fisher D.M., Segredo V., Caldwell J.E., Bragg P., Sharma M.L., Gruenke L.D., Miller R.D. (1992). Pharmacokinetics of Rocuronium Bromide (ORG 9426) in Patients with Normal Renal Function or Patients Undergoing Cadaver Renal Transplantation. Anesthesiology.

[29] Matteo R.S., Ornstein E., Schwartz A.E., Ostapkovich N., Stone J.G. (1993). Pharmacokinetics and Pharmacodynamics of Rocuronium (Org 9426) in Elderly Surgical Patients. Anesth. Analg..

[30] Jacob S., Nair A.B. (2018). Cyclodextrin complexes: Perspective from drug delivery and formulation. Drug Dev. Res..

[31] Clarke R.C., Sadleir P.H.M., Platt P.R. (2012). The role of sugammadex in the development and modification of an allergic response to rocuronium: Evidence from a cutaneous model. Anaesthesia.

[32] Limbird L.E. (1988). Receptors linked to inhibition of adenylate cyclase: Additional signaling mechanisms. FASEB J..

